# Brain vascular heterogeneity: implications for disease pathogenesis and design of in vitro blood–brain barrier models

**DOI:** 10.1186/s12987-018-0097-2

**Published:** 2018-04-23

**Authors:** Midrelle E. Noumbissi, Bianca Galasso, Monique F. Stins

**Affiliations:** Malaria Research Institute, Dept. Molecular Microbiology and Immunology, Johns Hopkins Bloomberg School of Public Health, 615 N. Wolfe Street, SPH East 4135, Baltimore, MD 21205 USA

**Keywords:** Blood–brain barrier, Cerebral vasculature, Grey matter, In vitro models, Neurodegeneration, Neurovascular unit, White matter

## Abstract

The vertebrate blood–brain barrier (BBB) is composed of cerebral microvascular endothelial cells (CEC). The BBB acts as a semi-permeable cellular interface that tightly regulates bidirectional molecular transport between blood and the brain parenchyma in order to maintain cerebral homeostasis. The CEC phenotype is regulated by a variety of factors, including cells in its immediate environment and within functional neurovascular units. The cellular composition of the brain parenchyma surrounding the CEC varies between different brain regions; this difference is clearly visible in grey versus white matter. In this review, we discuss evidence for the existence of brain vascular heterogeneity, focusing on differences between the vessels of the grey and white matter. The region-specific differences in the vasculature of the brain are reflective of specific functions of those particular brain areas. This BBB-endothelial heterogeneity may have implications for the course of pathogenesis of cerebrovascular diseases and neurological disorders involving vascular activation and dysfunction. This heterogeneity should be taken into account when developing BBB-neuro-disease models representative of specific brain areas.

## Background: the blood–brain barrier as part of the neurovascular unit

The BBB separates the peripheral blood circulation from the brain parenchyma to allow for optimal functioning of the central nervous system (CNS). The actual barrier site is formed by CECs that line the cerebral vasculature and tight junctional (TJ) proteins that securely connect two adjacent CECs, thus limiting paracellular transport. Transport of substances across the BBB is generally dependent on the characteristics of the compound that crosses, such as lipid- versus water-solubility.

Lipid-soluble compounds can cross relatively easily into the brain: for example, certain drugs, e.g., anesthetics, drugs of abuse, and barbiturates, dissolve into the cell membranes of CECs and diffuse across the BBB into the CNS. Other compounds can cross the BBB via more specific transcellular pathways which include: (i) carrier-mediated transport of molecules, such as glucose and amino acids, (ii) receptor-mediated endocytosis and transcytosis of large macromolecules like transferrin, and (iii) adsorptive-mediated endocytosis and transcytosis of charged plasma proteins (see also reviews by Abbott et al.) [1, 2]. Additionally, paracellular transport occurs between two adjacent CECs, allowing for diffusion of compounds such as small, water-soluble compounds, e.g., ions and small hydrophilic solutes. The BBB also allows for trafficking of immune cells into the brain as a part of regular immune surveillance. Certain pathogens, such as human immunodeficiency virus (HIV), hijack these natural mechanisms and use immune cells to gain entry into the CNS; the so called “Trojan Horse” mechanism [3]. In general, immune cells cross the BBB via the paracellular pathway, but it is also possible for them to utilize transcellular pathways [1, 2, 4]. Together, all these transport and immune surveillance pathways play a major role in maintaining CNS homeostasis.

The BBB-endothelium is a key component of the NVU. The concept of the NVU states that in order to maintain CNS homeostasis, there is cross talk between the different cellular components of the NVU including neurons, astrocytes, glia, pericytes, and CECs [5]. Aberrant signaling due to infections or disease-mediated activation of any of its constituent components can lead to the disturbance of brain homeostasis and functioning [1, 6, 7]. Key functions of the NVU include maintaining the CEC phenotype, coupling blood flow to brain activity, linking neurogenesis to new blood vessel formation, and regulating cellular interactions between the vasculature, neurons, and glial cells (see also reviews by [5, 8–10]).

Astrocytes are specialized glial cells which function in maintaining a healthy CNS [11–20]. Within the NVU, astrocytes regulate CEC phenotype by increasing CEC barrier integrity [21–23] and enhancing TJ structures [24–27]. Additionally, astrocytes were shown to increase the expression of specific transporters on CECs such as Na–K–Cl and L-system amino acid transporters [28–30]. Factors involved in regulating CEC phenotypes include; glial cell-derived neurotrophic factor (GDNF) [31]; transforming growth factor (TGF)-β1 [32]; retinoic acid (RA) [33]; and vascular endothelial growth factor-A (VEGF-A) [34–36].

Pericytes are perivascular cells that wrap around capillaries in the brain and are part of the NVU [37]. They are characterized by the presence of smooth muscle actin fibers and therefore may play a role in local regulation of vasodilatation and constriction [38]. Pericytes have also been shown to increase the BBB integrity [39, 40] and the proportion of TJ proteins (occludin and claudin-5) [41]. A deficiency of pericytes, as seen in *Pdgfrb*^+/−^ mice, resulted in decreased capillary length [42] and a concomitant increase in BBB permeability due to an increase in transcytosis across CECs [43], decreased expression of TJ and scaffolding proteins (ZO-1, occludin, and claudin), and the adherens junction protein VE-Cadherin [42]. This change in brain vascular permeability was heterogeneous across the CNS: the highest increase occurred in the cortex, striatum, and hippocampus while there was a significantly lower change in permeability in the interbrain (or diencephalon), midbrain, and cerebellum [44, 45].

Besides astrocytes and pericytes that interact closely with the CECs, microglia also affect the BBB-endothelial phenotype and function. Microglia are derived from the mesodermal lineage and migrate into the CNS early in embryonic development to become the resident immune cells of the brain [46–48]. Upon activation by, for example, microbial infections or traumatic brain injury, microglia can differentiate into the pro-inflammatory M1 or anti-inflammatory M2 phenotypes with a concurrent morphological shift from small cell bodies with long processes to enlarged amoeboid-like cells [49]. The M1 microglia promote BBB opening by secreting pro-inflammatory cytokines, such as interleukin-1 β (IL-1β), tumor necrosis factor-α (TNF-α), and nitric oxide (NO). In contrast, M2 microglia promote immunosuppression via release of TGF-β and angiogenesis through VEGF release in tumors [49]. VEGF from microglia were also shown to enhance BBB permeability via downregulation of ZO-1 [6, 50–52].

It should be noted that CECs can also reciprocally influence the functioning of its neighboring cells within the NVU [5, 10, 53]. Guo et al. [54] demonstrated that secretion of brain-derived neurotrophic factor (BDNF) by CECs was vital for neuroprotection. In addition, soluble factors secreted by CECs were found to enhance the proliferation of oligodendrocyte progenitor cells (OPC); which are precursors of oligodendrocytes as well decrease apoptotic OPC death in vitro [55, 56]. Furthermore, extracellular vesicles derived from rat brain CECs were shown to have a role in promoting OPC survival, proliferation and motility in a dose-dependent manner [57]. However, the mechanisms underlying these effects are not well understood.

Another important part of the NVU is the extracellular matrix (ECM), which also contributes to specific CEC phenotypes and functions [58, 59]. ECM refers to the non-cellular component of the NVU deposited in the space between CECs, pericytes, and astrocytic end-feet. The ECM is composed of a mixture of proteins, including different collagens, laminins, fibrillins, fibronectin, and vitronectin. CECs, astrocytes, and pericytes deposit various isoforms of laminin (α_2_ and α_4_) in ECM; these play a pivotal role in regulating BBB integrity [60–63]. Laminin-10 was shown to promote repair in an in vitro model of BBB hypoxic injury [64]. Furthermore, extracellular matrix proteins such as heparan sulfate proteoglycans (HSPG), perlecan, collagen IV [65, 66], and integrin-matrix interactions [67, 68] have been implicated in regulating BBB integrity. Collagen type IV alpha 1 (COL4A1) and collagen type IV alpha 2 (COL4A2) are the most abundant form of collagen IV in ECM proteins [69]. COL4A1 and COL4A2 are highly conserved in humans and mutations in one or both of them have been linked to various organ diseases, including cerebral diseases, such as porencephaly and cerebrovascular/intercerebral hemorrhages [70–87].

Regulatory enzymes, such as matrix metalloproteinases (MMPs) are also associated with the ECM [88–92]. Both MMPs and tissue inhibitors of metalloproteinases (TIMPs), are secreted by CECs, astrocytes, and pericytes and are involved in modifying the ECM and in the regulation of BBB integrity [93, 94]. For example, pericytes can regulate CE-derived MMP-9 production and in the absence of pericytes, there is a decrease in MMP-9 levels resulting in increased trans-endothelial electrical resistance (TEER) of CEC monolayers [94].

## Heterogeneity in cellular composition of the brain and its influence on the BBB

The human brain is divided into three main regions, each with distinct functions. These include the brainstem, which regulates automatic functions such as breathing and digestion, the cerebellum, which coordinates muscle movement and balance, and the cerebrum which is involved in higher functions such as learning and interpreting speech and touch. The brain is also segregated into cortical grey matter (GM) and white matter (WM); where the cellular composition differs considerably between GM and WM [95–97].

**Fig. 1:**
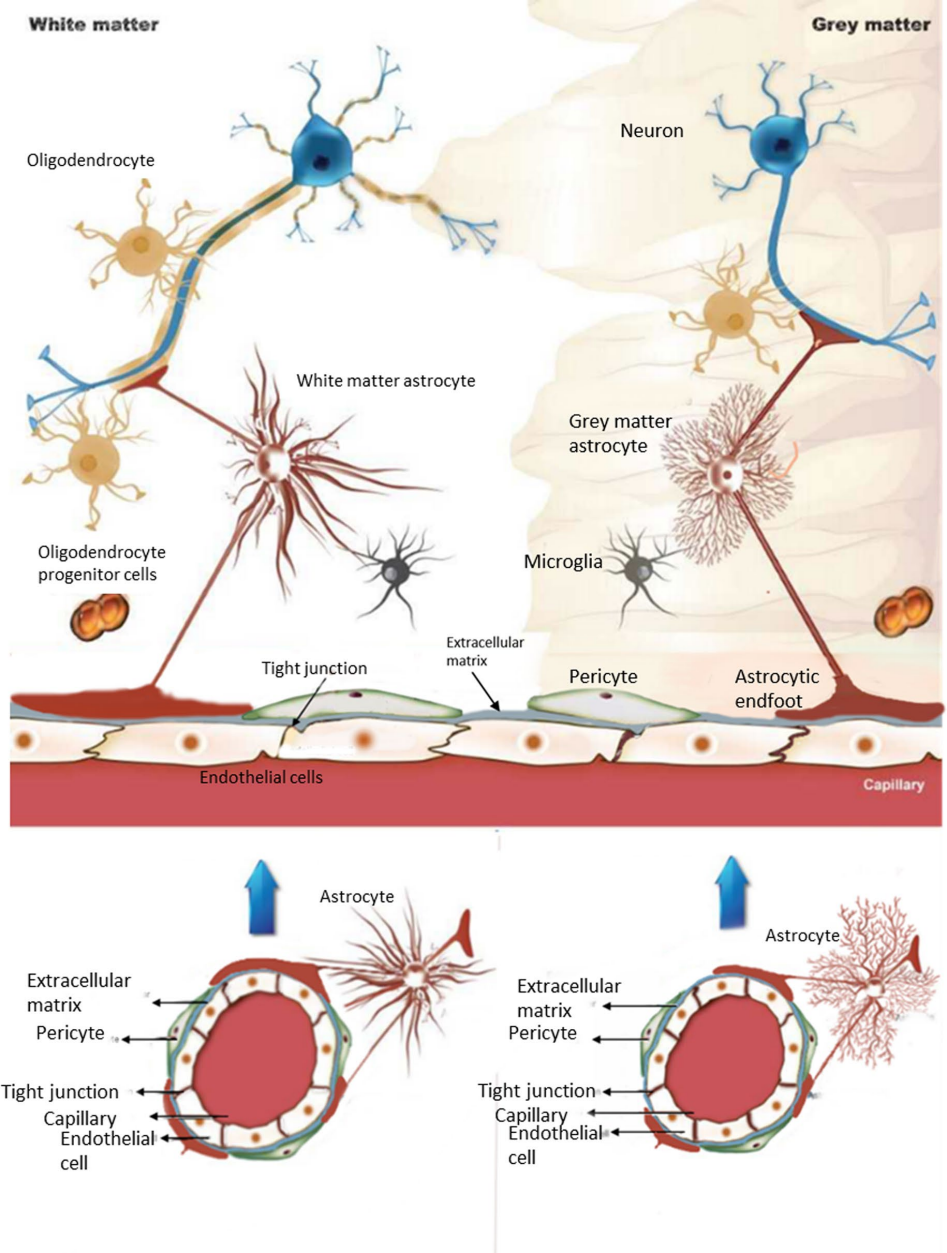
General representation of structural and cellular differences between GM and WM: the brain is approximately segmented in equal volumes of GM and WM, where the cellular composition differs considerably. GM has a high non-myelinated neuronal content and lesser extent of myelinated axons. WM is composed of both myelinated and non-myelinated axons; with higher myelin content is responsible for its whitish appearance. Similarly, GM and WM also exhibit differences in other resident brain cell types, including astrocytes, glial cells in number and morphology. These differences in the immediate environment of the vasculature may confer specific differential WM and GM vascular phenotypes that may be reflected in amount and organization of tight junctions, expression of various receptors, transporters and responses to stimuli in neurovascular diseases

Although GM and WM have approximately equal volumes [98], the ratio of non-neuronal to neuronal cells is 1.5:1 in the cortical GM compared to 15:1 in the cortical WM [95]. The GM has a higher neuronal content including neuronal cell bodies, dendrites, non-myelinated axons, to a lesser extent of myelinated neurons, and glial cells, including resident astrocytes [11, 96]. The WM is predominantly composed of both myelinated and non-myelinated axons, astrocytes, and myelin-producing glia [96, 97]. Additionally, there are differences in morphology between astrocytes residing in GM and WM. GM astrocytes, traditionally called protoplasmic astrocytes, display stem branches with many branching processes, while in WM, astrocytes exhibit more fiber-like processes [11] (Fig. 1). Advancement of imaging techniques and investigations into their physiology have led to the use of a variety of names for these different astrocytes to better reflect individual characteristics. Despite that, currently there is no uniform astrocyte nomenclature [99]. Differential characteristics include higher levels of vimentin, nestin and glial fibrillary acidic protein in astrocytes derived from WM than from GM [99]. Although there is equal distribution of glucose transporter-1 (GLUT-1) 52 kDa isoform across the GM and WM astrocytes [100], the GLUT-1 45 kDa isoform is mainly observed on the GM astrocytes [101]. This suggests that besides phenotypic differences between astrocytes residing in GM versus WM, functional differences are also present. Taken together, these functional astrocyte differences between WM and GM, in turn, may affect brain vasculature in these different areas. However, the molecular mechanisms whereby these various astrocytes affect the CEC characteristics in these brain areas are unclear [99].

Differences in both density and orientation also exist between the GM and WM vasculature. Most apparent is the higher blood vessel density in GM than that in WM [102]. Also, the arrangements of blood vessels differs: cerebellar GM vessels are arranged perpendicular to the pyramidal cell layer whereas the WM vessels are longer and oriented parallel to axonal fibers [102].

In addition to variances in general structure and organization of the brain vasculature, there are differences in endothelial barrier function between anatomical regions. Apart from a high permeability in the vasculature of the Area Postrema and choroid plexus, there are differences in the molecular composition of the vascular junctional proteins that may reflect differences in functionality of the BBB. For example, expression of occludin, claudin-5, and adherens junction α-catenin is higher in WM compared to GM [96]. Accordingly, there is a higher barrier function seen in primary CEC cultures derived from WM compared to those from GM [96]. Moreover, cytoskeletal structural differences in the vasculature between WM and GM were demonstrated [103]. Interestingly, astrocytic end feet expressed lower levels of GFAP in GM compared with WM and this correlated with a higher tendency of hemorrhage in the GM vasculature [103].

Apart from these differences in the vasculature of the WM and GM, CEC gene expression also differs along the length of the cerebral vasculature tree (arterioles, capillaries, and venules) [104, 105]. For example, CEC gene expression related to solute transport, e.g., monocarboxylate transporter 1 and plasma membrane Ca^2+^ ATPase Type 2, were significantly increased in capillaries compare to venules [105]. Differences also exist between cerebral and pial (of pia mater origin) microvessels in the brain [106–108]. Even though these micovessels share some common characteristics [106], pial microvessels lack envelopment by astrocytic end feet resulting in a diverse appearance of tight junctions and endothelial barrier antigens [108]. Similarly, differences in the expression of astrocyte dependent enzymes such as γ-glutamyl transpeptidase (GGTP) and alkaline phosphatase (AP) [107] were observed, such as absence of expression of GGTP in rat pial microvessels compared to a strong expression in cerebral vessels [107]. In summary, these studies clearly demonstrate significant heterogeneity in the cerebral vasculature, as well as differences in the composition of NVU components between the GM and WM that may be related to differential CEC gene expression along the vascular tree.

## Association of brain vasculature with GM and WM neuropathologies

Considering the existence of structural and functional variations in the cerebral vasculature between various regions of the brain, it is imperative to consider these vascular differences when evaluating neuropathologies. Here, we will briefly discuss select neuropathologies that involve an activation and/or dysfunction of the brain vasculature and its association with regional pathology, namely multiple sclerosis (MS), schizophrenia, HIV-associated neurocognitive disorders (HAND), and cerebral malaria (CM).

MS is an autoimmune disease of the CNS and its widely studied neuropathology exhibits region-specific differences in the brain. MS presents with areas of focal neuronal demyelination, axonal loss, immune cell infiltration, and involvement of the BBB [109–114]. MS lesions usually develop alongside brain vasculature and involve disruption of the BBB structure and function [115]. MS presentations differ from GM, as lesions in the WM involve disruption of the BBB leading to immune cell infiltration into the CNS, whereas this is not always noted in lesions in the GM vasculature [116, 117]. Furthermore, the cuprizone experimental mouse model of MS also shows increased BBB activation in WM compared to the GM [118–120]. These differences in the representation of MS in the brain GM and WM is most likely due to the inherent differences in the brain GM versus WM vasculature.

Schizophrenia, a neuropsychiatric disorder, is characterized by significant brain abnormality and regional variability, including involvement of WM pathologies, especially those involving frontal, fronto-temporal, and fronto-limbic connections [121–124]. Also here, regional structural differences have been observed, e.g., WM myelin disturbance, deterioration of the neuropil, loss of synaptic connectivity, and functional impairment of oligodendrocytes [125, 126]. Postmortem brain studies have reported higher expression of pro-inflammatory cytokines like IL-6, TNF-α, and transcription factor NF-κB in the WM of the frontal cortex compared to the GM [127–129]. In individuals with first-episode schizophrenia, signs of axonal degeneration appeared only in the focal areas of frontal lobe WM areas [130]. Similarly, in patients with new-onset schizophrenia, WM inflammation was associated with elevated serum S100B levels, implicating WM inflammation coupled with BBB hyper-permeability [131]. A recent study by Greene et al. [132] was the first to use molecular-based evidence to show involvement of the vasculature and BBB alterations in schizophrenia. Thus, the existence of vascular heterogeneity may contribute to the differential presentation of schizophrenia pathologies in GM versus WM regions of the brain.

In HIV-1 infected patients, the virus can enter into the CNS in the early stages of infection, eventually leading to neurocognitive impairments, including HIV-associated neurocognitive deficits (HAND). Both in vitro and in vivo studies have demonstrated activation of the brain endothelium and functional impairment of the BBB, including upregulation of cell adhesion molecules, downregulation of TJ complex components, and enhanced passage of immune cells across the BBB into the CNS, resulting in “cuffing” [133, 134]. Brain autopsy studies also showed a correlation of the severity of HAND with WM degeneration and gliosis [135, 136]. Interestingly, the brain vasculature appears more compromised in WM, as immunostaining for BBB junctional molecules, such as occludin and ZO-1, was either absent or more fragmented in the WM than in the GM [137]. Because of the brain vasculature’s involvement in HAND, brain vascular heterogeneity is also very likely to play a role in the manifestation of the differential pathologies in various brain regions,

Another infectious disease involving brain vascular inflammation/activation is CM, a severe neurological complication resulting from infection with the *Plasmodium falciparum* parasite. The hallmark of CM is sequestration of *P. falciparum*-infected red blood cells (Pf-IRBC) inside the vasculature, which leads to the activation of the BBB, as shown by increased ICAM-1 expression and decreased junctional markers [138–143]. Postmortem studies of brains from human CM patients show marked pathological differences between WM and GM. Highly apparent is the abundance of hemorrhagic punctae in the WM, associated with increased fibrin accumulation [144–147]. These differences in pathologies could be related to differences in the vasculature between WM and GM.

As shown previously and outlined above, there are clear differences in protein expression along the vascular tree [148]. However, limited information exists in terms of BBB physiology and EC phenotype in different regions of the brain [96, 99, 148] and how this relates to neuro-disease pathogenesis. Taken together, the above discussed neurological conditions and infections underline existence of regional pathological differences in the WM versus the GM as well as the involvement of brain vasculature. Seemingly, more pathologies present in the WM than in GM areas. Therefore, we hypothesize that these observed brain vascular differences between the GM and WM areas significantly contribute to their differential pathologies.

## In vitro modeling of the BBB/NVU with relevance to neuropathologies

To study neuropathologies involving the vasculature of the brain, various in vitro BBB models have been developed, mostly from CEC isolated from GM areas. Initially, in vitro models of the BBB were single-cell cultures composed of a monolayer of primary CECs derived from either human, bovine, porcine, or murine sources [149–154]. Primary CECs have a limited lifespan and exhibit significant donor-to-donor variability, which can affect interpretation of experimental outcomes. To study human disease, human derived cells are also preferential. As a result of this, immortalized brain EC lines, such as human derived hCMEC/D3, were developed as alternatives to primary CECs [155–159], but these cells still showed relatively low TEER. More recently, human pluripotent stem cell (hPSC)-derived CECs have been employed as a potential cell source and have shown significantly higher barrier integrity compared to the primary and immortalized cell lines when cultured in the presence of retinoic acid (RA) [154, 160–166]. Although the high barrier resistance could suggest BBB phenotype for the hPSCs, a more epithelial phenotype cannot be excluded. Besides TEER, other markers have been evaluated, though in a limited fashion. De Stefano et al. [167] demonstrated that there were no significant morphological changes in both hPSC-derived CECs and immortalized human CECs in response to fluid shear stress. They also showed that shear-induced motility was significantly reduced in hPSC-derived CECs [167]. However, these findings do not demonstrate whether these cells are representative of GM or WM vasculature and additional markers would need to be tested to fully validate the hPSCs for BBB modeling.

As outlined above, various cellular components of the NVU influence CEC phenotype and cerebrovascular integrity. In the earlier models, other cellular NVU components were not incorporated and there was minimal consideration of environmental influences, such as blood flow and pressure [149, 168]. To further improve specific BBB characteristics, co-culturing with cellular components of the NVU, specifically astrocytes or pericytes, as well as the addition of astrocyte- or pericyte-conditioned medium, has been utilized [21, 39, 40, 169–180]. Selection of appropriate culture media in co-culture experiments has been shown to be an important factor influencing the BBB integrity [181–183]. Several studies have demonstrated that the direct CEC environment, e.g., cell–cell contact, soluble factors or extracellular vesicles, is critical for the development and maintenance of the BBB properties and thus influence the cellular function within the NVU [6, 23, 41, 184]. Therefore, in order to obtain a physiologically relevant in vitro BBB model, the effect of these influences from within the NVU and how this may affect the BBB functionality should be considered, including a WM versus GM environment.

Physical factors and mechanical forces, such as shear stress and cyclic strain due to flowing blood, also affect endothelial structure and physiology [185, 186]. In vitro studies demonstrated that flow improved the barrier integrity of CECs [32, 187–190]. In contrast, supra-physiological shear stress and pulsatile flow can lead to the deterioration of BBB integrity [191]. Loss of blood flow promoted cytokine release (IL-1β, IL-6, and TNF-α) which, in turn, mediated a decrease in TEER, resulting in BBB leakage [192]. Additionally, both substrate elastic modulus [193–195] and ECM composition [196, 197] affect endothelial responses, including cytoskeletal realignment, inflammation, and cell morphology, to shear stress. When designing an in vitro model of the BBB, substrate curvature (flat or curved surfaces) and culture dimensionality should also be considered. Ye et al. [198] demonstrated that changing the substrate curvature from flat to curved resulted in a change in cell orientation of the CECs [198]. Additionally, three-dimensional in vitro BBB models were shown to restrict viral infections compared to the two-dimensional models [199]. Together, these studies demonstrate the numerous factors that may need to be considered when designing a physiologically relevant in vitro BBB model.

It is very challenging to incorporate all of the aforementioned factors that influence the BBB. Advancements in technology have provided additional options for in vitro BBB modeling to better mimic the BBB biophysical environment, e.g., addition of flow and dimensionality/curvature such as lab on a chip models [200]. They also allow for the controlled application of inflammatory stimuli to study the responses of various brain cell types during neuroinflammation [201–203]. Utilizing the BBB-on-chip model, the TEER of CECs cultured on microfluidic chips (36.9 Ω cm^2^) was higher than that of CECs grown on Transwell™ chambers (28.2 Ω cm^2^) [203]. Similar technology has also been used to create a NVU on a chip by co-culturing CECs with pericytes, neurons, and astrocytes in a three-dimensional collagen I matrix [201]. Recently, cylindrical collagen gels were generated using the “viscous fingering” method within 3D BBB chips composed of CECs, pericytes, and astrocytes. Notable findings include that (1) the CECs generated an abluminal basement membrane and (2) the astrocytes in combination with CECs significantly reduced the permeability (a phenomenon that was not observed with EC-pericyte co-culture) [202]. Additionally, a recent study used a sophisticated triple BBB co-culture of a human brain endothelial cell line with primary astrocytes and brain pericytes assembled in a poly(dimethylsiloxane)-based chip that also allowed for simultaneous assessment of flow, morphology, TEER, and permeability measurements [204].

Thus, advantages of the BBB-on-chip include: (i) incorporation of flow (exposure of CECs to shear stress); (ii) culture of CECs in a three-dimensional environment; and (iii) measurement of physiological functions (such as permeability and TEER) in real-time along with fluorescence imaging of cell–cell junctions [203, 205]. Although current technical advances allow for the development of organ-on-chip systems, this technology is highly specialized and requires specialized facilities for fabrication of these devices. The major disadvantage of these lab-on-a-chip systems is their limited commercial availability and high price point [200]. To our knowledge, at this point, no (affordable) commercial system is available that incorporates all the desired parameters.

In summary, these studies outline important design considerations for creating in vitro models of the BBB. Additionally, they demonstrate recent advancements in technology that may be used to model the regional heterogeneity of the brain parenchyma. A key assumption of most in vitro model designs is that the BBB/CEC phenotype is homogeneous across the brain. However, as discussed above, phenotypic differences exist along the vascular tree [105, 148] and in varying brain regions [44, 96]. This, coupled with the knowledge that certain neuropathologies differentially affect WM and GM, should prompt development of more representative in vitro models of the BBB that can better mimic the particular in vivo environment of the neuropathological condition, e.g., culturing CEC with either WM or GM characteristics. Exposing these CEC’s to their appropriate brain environment may confer and/or approximate these characteristics and provide a better option for BBB- neurodisease modeling.

## Conclusions and future directions

Regional cellular heterogeneity in the brain parenchyma may contribute to the differences in CEC phenotype within the cerebral microvasculature. The two major regions of the brain, the GM and WM, have distinct vascular patterns, cellular compositions, and molecular phenotypes. These relative superficial differences in the brain vasculature warrant deeper investigation of the specific regional variability of the BBB. A comprehensive analysis of molecular phenotypes and functional differences of the vasculature between brain regions would allow for better understanding of diverse neuro-pathologies. This may have further implications for the design of better and more targeted therapeutic interventions in neurovascular diseases. In vitro BBB modeling offers possibilities for targeted and controlled assessment of BBB pathogenesis but, thus far, primarily relies on the assumption of homogeneity of the BBB across the various brain regions. In this review, we have highlighted some region-specific differences in the BBB and propose design considerations for developing more representative models of the BBB by incorporating these regional heterogeneities. A more comprehensive in vitro BBB design should also encompass the region-specificity of the NVU- brain milieu for it to translate effectively to different neurovascular disease conditions of interest.

## Abbreviations

ACM: astrocyte-conditioned medium
BBB: blood–brain barrier
CEC: cerebral microvascular endothelial cells
CM: cerebral malaria
HAND: HIV-associated neurocognitive disorders
HSPG: heparan sulfate proteoglycans
NVU: neurovascular unit
TEER: trans-endothelial electrical resistance
TGF: transforming growth factor
TIMP: tissue inhibitors of metalloproteinases
MMP: matrix metalloproteinases
CNS: central nervous system
HIV: human immunodeficiency virus
TJ: tight junction
VEGF: vascular endothelial growth factor
NO: nitric oxide
IL: interleukins
TNF-α: tumor necrosis factor-α
P-gp 1: p-glycoprotein 1
γ-GTP: *gamma*-glutamyl transpeptidase
GLUT-1: glucose transporter-1
OPC: oligodendrocyte progenitor cell
NF-κB: nuclear factor kappa-light-chain-enhancer of activated B cells
ZO: zonula occludens proteins
JAM: junctional adhesion molecules
